# Differential Response of Two Tomato Genotypes, Wild Type cv. Ailsa Craig and Its ABA-Deficient Mutant *flacca* to Short-Termed Drought Cycles

**DOI:** 10.3390/plants10112308

**Published:** 2021-10-27

**Authors:** Bojana Živanović, Sonja Milić Komić, Nenad Nikolić, Dragosav Mutavdžić, Tatjana Srećković, Sonja Veljović Jovanović, Ljiljana Prokić

**Affiliations:** 1 Institute for Multidisciplinary Research, University of Belgrade, Kneza Višeslava 1, 11030 Belgrade, Serbia; bojana.zivanovic@imsi.rs (B.Ž.); sonjamilic@imsi.rs (S.M.K.); nnikolic@imsi.bg.ac.rs (N.N.); gane@imsi.rs (D.M.); tatjanas@rcub.bg.ac.rs (T.S.); 2 Center for Green Technologies, Institute for Multidisciplinary Research, University of Belgrade, Kneza Višeslava 1, 11030 Belgrade, Serbia; 3 Faculty of Agriculture, University of Belgrade, Nemanjina 6, 11080 Belgrade, Serbia

**Keywords:** abscisic acid, drought, recovery period, cell wall constituents, stress memory, tomato mutants, stomatal sensitivity

## Abstract

Two tomato genotypes with constitutively different ABA level, *flacca* mutant and wild type of Ailsa Craig cv. (WT), were subjected to three repeated drought cycles, with the aim to reveal the role of the abscisic acid (ABA) threshold in developing drought tolerance. Differential responses to drought of two genotypes were obtained: more pronounced stomatal closure, ABA biosynthesis and proline accumulation in WT compared to the mutant were compensated by dry weight accumulation accompanied by transient redox disbalance in *flacca*. Fourier-transform infrared (FTIR) spectra analysis of isolated cell wall material and morphological parameter measurements on tomato leaves indicated changes in dry weight accumulation and carbon re-allocation to cell wall constituents in *flacca*, but not in WT. A higher proportion of cellulose, pectin and lignin in isolated cell walls from *flacca* leaves further increased with repeated drought cycles. Different ABA-dependent stomatal closure between drought cycles implies that acquisition of stomatal sensitivity may be a part of stress memory mechanism developed under given conditions. The regulatory role of ABA in the cell wall restructuring and growth regulation under low leaf potential was discussed with emphasis on the beneficial effects of drought priming in developing differential defense strategies against drought.

## 1. Introduction

Water scarcity has become one of the greatest problems for agricultural production, with rapidly increasing instances in many areas of the world. Drought is identified as a major threat to crop production causing billions in annual economic losses, mainly from the agricultural sector [1,2]. The impact of drought on plants depends not only on the genotypes, the developmental progression of specific tissues and of the whole plant system but also on the duration, frequency and intensity of drought, as well as the presence of other abiotic stresses, such as high temperature, sunlight intensity or UV radiation [3,4,5]. Plants respond to drought through morphological, physiological and biochemical modulations controlled at the molecular level by hormones, sugars, proline and reactive oxygen species (ROS) via a complex network, i.e., different signaling pathways leading to a regulated gene expression [6,7,8,9,10,11]. Plants eliminate elevated ROS by an efficient antioxidative defense system consisting of the enzymatic and non-enzymatic antioxidants [12,13]. Maintenance of the ascorbate redox state is important for plants’ homeostasis under water deprivation [14]. Furthermore, plant cell wall composition and integrity plays an important role in abiotic stress tolerance and is one of the important traits in the selection of drought tolerant varieties [15,16].The drought and salt-induced perturbation of the cell wall, which modifies its components’ synthesis, are well documented [17,18,19].

Stress imprinting (or priming), which positively affects plant survival under repetitive stresses, is widely accepted as an alternative agriculture measure to prepare plants to withstand drought episodes accompanied with high insolation and heat waves [20,21]. Previous studies have shown that plants pre-exposed to multiple drought stress episodes exhibit rapid and more efficient responses compared to plants that experience drought for the first time [21,22,23]. Drought priming can reset plant metabolism leading to changes in primary and secondary metabolism, forming a short term memory based on the accumulation of carbohydrates, amino acids [24] and antioxidants [25].

Tomatoes (*Lycopersicon esculentum* Mill.) are one of the most important and widely grown vegetables in the world, especially in the Mediterranean region [26,27]. Its high nutritional value is based on a high content of phytonutrients, such as phenolic compounds and carotenoids [28]. Due to its short lifecycle and relatively small genome, the tomato is an excellent fleshy fruit model system [29]. Tomato plants are very sensitive to drought, which is a consequence of their limited tolerance against this stress [30]. The proteomic analyses of tomato plants exposed to drought and their recovery period have identified numerous drought responsive proteins involved in the antioxidative defense (Cu/Zn superoxide dismutase, catalase, ascorbate peroxidase), photosynthetic metabolism, electron transport (NADP reductase), protein biosynthesis and cell wall metabolism [29,30,31]. It was shown by using gene ontology enrichment analyses that drought affects the regulation of histone encoding genes, chlorophyll binding, heat shock proteins and genes that are related to protein products involved in the cell wall and sugar metabolism, as well as ABA-controlled chloroplast to nucleus signaling [29,32].

Abscisic acid (ABA) is a key molecule that underpins the regulatory mechanisms of a plant’s response to drought, such as the regulation of the turgor pressure of guard cells and stomatal closure, maintenance of water balance, osmotic stress tolerance and activity of antioxidative enzymes [33,34,35]. In plants exposed to progressive soil drying, significant changes in ABA and/or leaf water potential (Ψ), as chemical and hydraulic signals, are responsible for stomatal closure, implying their mutual interaction in the regulation of the stomatal aperture [36,37]. Turgor changes and stomatal closure under stress conditions can also occur due to modulation of the elastic properties of the cell wall and the activity status of the anion channels in guard cells [38].

Recent studies have demonstrated that ABA may contribute to increased drought tolerance related to drought memory effect [39,40]. The stomata of plants, which have once been exposed to drought stress, may also remain partially closed during the recovery period. Thus, partially closed stomatal contribute to the reduction in water loss during subsequent drought episodes [22]. Stomatal closure is associated with increased ABA content and ABA-related genes expression, which are key modulators of ABA-biosynthetic and catabolic pathways [41].

Elevated ABA content under drought is predominantly the result of de novo biosynthesis of genes included in ABA metabolism, such as 9-cis-epoxycarotenoid dioxygenase (*NCEDs*) [42,43]. Moreover, it can also be the result of the decreased ABA catabolism [42] and/or ABA redistribution induced by pH fluctuations [44].

In this study, we used one of the tomato (Ailsa Craig cv.) ABA-deficient mutants, *flacca*, which, compared to wild type, accumulates a lower content of ABA [45].The last step in ABA biosynthesis involves the oxidation of abscisic aldehyde to ABA by the molybdenum containing aldehyde oxidase (AO; EC 1.2.3.1). In *flacca,* due to the deletion of six base pairs in a molybdenumcofactor (Mo-Co) sulfurase, sulphuration of Mo-Co in AO is inhibited and, therefore, their oxidative activity is suppressed [45,46]. These ABA-deficient mutants wilt faster due to their higher transpiration rates and abnormal stomatal behavior, possess a reduced leaf area, thinner stem, more expressed epinasty and diminished aerial root formation [46,47,48,49,50]. Interestingly, upon treatment with exogenously applied ABA the mutant plants’ phenotype characteristics were reversed to wild type [47,48,49].

In this work, we compare a plant’s response to repeated drought cycles (watered/drought-induced/re-watered plant status) during the vegetative development of two tomato genotypes, differing in the constitutive leaf ABA level, with the final aim to explore the ABA role in short term stress memory and acclimative mechanisms to water deficit. Recently, we reported that the same *flacca* mutant exhibited constitutively higher levels of soluble sugars and free amino acids (AAs) compared with its parent line Ailsa Craig cv. [51]. Here, we analyze the drought effects on plants by imposing more natural conditions on our model system that would allow maximal photosynthetic rates and faster growth. Furthermore, comparison of the molecular and physiological processes induced by repeated cycles of moderate drought might contribute to elucidation of the underlying mechanisms of plant stress memories and drought tolerance. We also compared a plant`s status following prolonged recovery after one drought cycle with recovery after three repeated drought cycles in order to determine whether different patterns of drought tolerance emerge. Additionally, an understanding of the genotype-specific molecular basis of acclimatization response to drought could help us to induce specific traits in plants during the growing season, which could be beneficial under subsequent drought.

## 2. Results

### 2.1. Drought Cycles

#### 2.1.1. ABA Content, Stomata and Water Status

**Figure 1:**
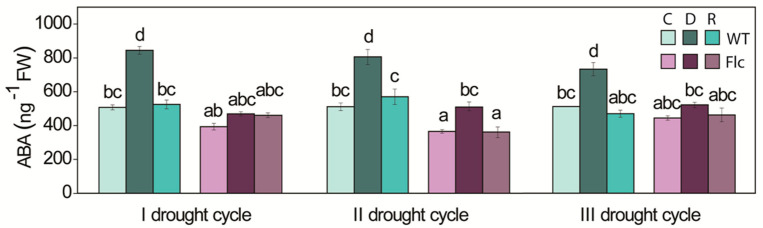
ABA content in the leaves of WT and *flacca* tomato genotypes subjected to three drought cycles (D), followed by three days of re-watering (R), with respective control plants (C). Values are presented as means ± SE (*n* ≥ 7). Different letters denote significant differences between means according to Tukey HSD post hoc test *p* ≤ 0.05.

Leaf ABA content in both genotypes did not vary throughout the growth period under well watered conditions, with 20–30% less ABA concentration in the ABA-deficient mutant of Ailsa Craig, *flacca*, compared to its parent line (Figure 1, Supplementary Table S1, genotype effect *p* = 0.000000). Water withdrawal to the same soil water content (SWC) during 6 days in the 1st drought cycle induced a 65% and 15% increase in ABA concentration, Figure 1. The next two drought cycles led to an ABA elevation of 57 and 43% in WT and 39 and 20% in *flacca*, respectively. In all three drought periods, ABA accumulation in *flacca* was similar to the values measured in turgid WT leaves. While the ABA content in WT bounced back after three days of rehydration to levels found prior to water scarcity in all three applied cycles; the same was obtained only in the 2nd and 3rd cycles in *flacca*.

**Figure 2:**
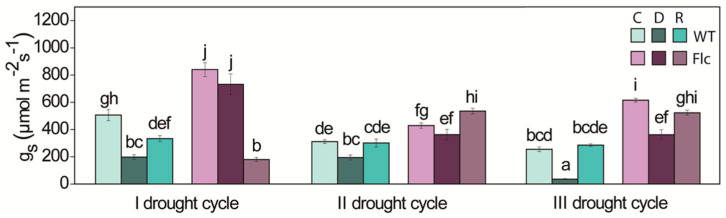
Stomatal conductance in the leaves of WT and *flacca* tomato genotypes subjected to three drought cycles (D), followed by three days of re-watering (R), with respective control plants (C). Values are presented as means ± SE (*n* ≥ 7). Different letters denote significant differences between means according to Tukey HSD post hoc test *p* ≤ 0.05.

Under optimal watering, the *flacca* mutant showed significantly higher stomatal conductance in comparison to WT plants throughout the whole period, with the least measured difference found in the 2nd cycle (Figure 2, Supplementary Table S1, genotype effect *p* = 0.000001). Stomatal conductance in WT drought-stressed plants varied in inverse proportion to the ABA accumulation, with the strongest response in the 3rd cycle. However, a statistically significant, but much smaller decrease compared to WT in the stomatal conductance, was also observed in *flacca* in the 2nd and 3rd drought episodes (Figure 2). Furthermore, stomata were more responsive to water status upon repeated re-watering treatments: stomatal conductance was restored to control values in the 2nd and 3rd drought episodes, while in the first cycle stomata remained partly closed in both genotypes (Figure 2).

**Figure 3:**
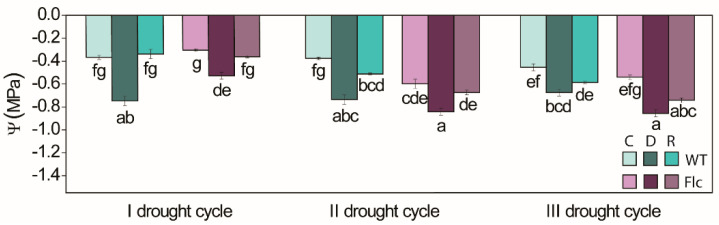
Leaf water potential (Ψ) in the leaves of WT and *flacca* tomato genotypes subjected to three drought cycles (D), followed by three days of re-watering (R), with respective control plants (C). Values are presented as means ± SE (*n* ≥ 7). Different letters denote significant differences between means according to Tukey HSD post hoc test *p* ≤ 0.05.

Leaf water potential (Ψ) under well watered conditions did not differ between genotypes; however, in the 1st drought this decreased to a larger extent in WT than in *flacca*. In the next two successive drought cycles, the reduction in leaf water potential in WT was slightly smaller than in *flacca*. After three days of re-watering, the leaf water potential fully recovered only in the 1st drought cycle, while it partly recovered in the 2nd and 3rd cycle (Figure 3).

#### 2.1.2. Drought-Induced Changes in Proline Content

**Figure 4:**
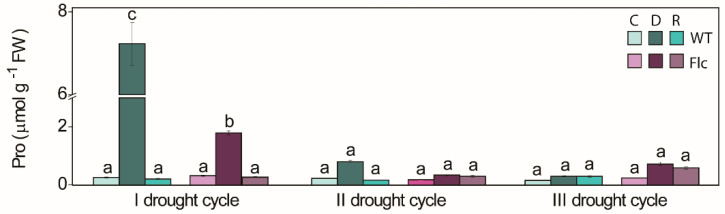
Proline content in the leaves of WT and *flacca* tomato genotypes subjected to three drought cycles (D), followed by three days of re-watering (R), with respective control plants (C). Values are presented as means ± SE (*n* ≥ 7). Different letters denote significant differences between means according to Tukey HSD post hoc test *p* ≤ 0.05.

Proline accumulation, as one of the most common plant responses to water deficit, was observed in both genotypes but to a much greater extent in WT (Figure 4). In the 1st drought cycle, proline concentration in WT increased more than 25 times and in *flacca* more than 7 times compared to turgid leaves. An initial proline concentration was reset upon re-watering, in the 1st cycle in both genotypes and in the 2nd cycle only for WT. In the other two drought episodes, proline content did not change in comparison to respective controls (Figure 4, Supplementary Table S2).

#### 2.1.3. Drought-Induced Changes in Ascorbate Redox State

In all three cycles drought induced an increase in total ascorbate content only in *flacca* plants, while in WT it remained unchanged. In *flacca*, the most intensive ascorbate rise was observed in the first drought episode, which was accompanied by the most prominent decrease upon re-watering. On the other hand, in the other two cycles, re-watered plants retained total ascorbate pull similar to water-stressed plants.

**Figure 5:**
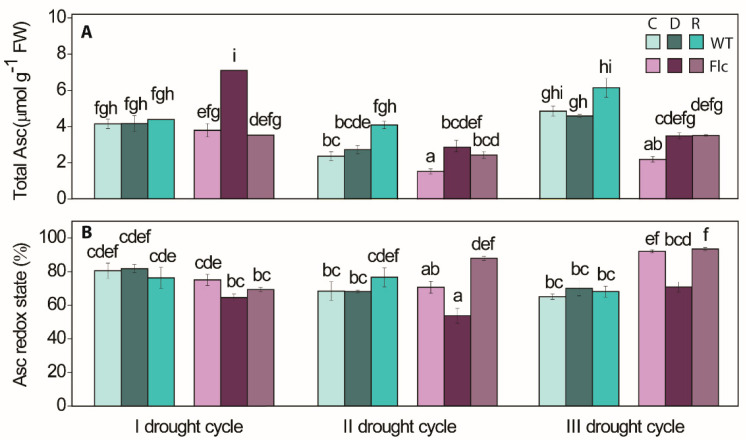
Total ascorbate content (**A**) and ascorbate redox state (**B**) in the leaves of WT and *flacca* tomato genotypes subjected to three drought cycles (D), followed by three days of re-watering (R), with respective control plants (C). Values are presented as means ± SE (*n* ≥ 7). Different letters denote significant differences between means according to Tukey HSD post hoc test *p* ≤ 0.05.

Moreover, in WT plants in the recovery phase, the total ascorbate content was higher only in the 2nd cycle, while in the 1st and 3rd it was similar to control and stressed plants. The ascorbate redox state in WT plants in all drought cycles was unchanged, while in *flacca* plants a slight decrease in droughted plants was noticed in comparison to controls and recovered plants (Figure 5).

#### 2.1.4. Drought-Induced Changes in ABA-Responsive Genes Expression

**Figure 6:**
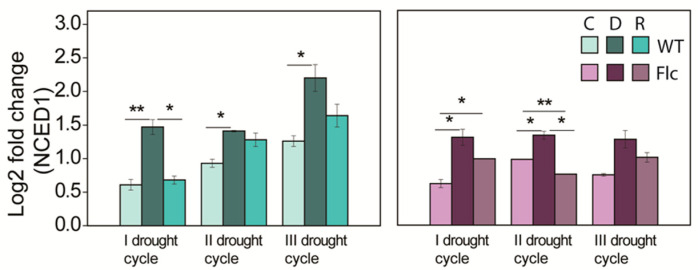
NCED1 expression in the leaves of WT and *flacca* tomato genotypes subjected to three drought cycles (D), followed by three days of re-watering (R), with respective control plants (C). Relative expression was determined by qRT PCR, and normalized to the housekeeping gene and calculated relative to WT start control. Values are presented as means ± SE (*n* ≥ 4). Asterisks denote significant differences between control, drought and recovered plants within individual genotypes in each drought cycle, according to Welch *t*-test (* *p* < 0.05, ** *p* < 0.01).

Drought treatment, in all three cycles, induced a significant increase in the expression of the *NCED1* gene in both genotypes, WT and *flacca*, though to a larger extent in WT plants. A maximal up-regulation of *NCED1* was obtained in the 3rd cycle in WT plants, reaching values four times higher than in the control from the first drought cycle. A progressive increase in the *NCED1* expression after rehydration during successive drought cycles was noticed in WT but not in *flacca* (Figure 6).

### 2.2. Prolonged Recovery after First Drought and Recovery after Third Drought

#### 2.2.1. Effects of Prolonged Recovery after First Drought and Recovery after Third Drought on Growth Parameters in WT and *flacca*

To determine the effects of repeated drought episodes and prolonged recovery on plant growth, we compared growth parameters at the end of the vegetative stage in three groups of plants: (1) the control plants, which were continuously irrigated, (2) the plants that experienced one drought stress followed by 15 days period of re-watering, and (3) the plants that experienced three drought cycles.

When water deficit was imposed as an isolated event followed by 15 days of re-watering, both the leaf fresh weight and the leaf area of WT and *flacca* increased; however, the effect was more pronounced in the mutant (Table 1). The increase in leaf fresh weight was accompanied by a double increase in the dry weight of leaves and by a 30% decrease in SLA only in *flacca*. Stem dry weight was also higher by 40% at the end of experiment in *flacca* plants that were submitted to only one drought episode.

WT plants that experienced three successive drought cycles compared with controls or R_1_ plants exhibited growth retardation of leaves and a slight increase in stem biomass at the end of the experiment (Table 1). On the contrary, *flacca* showed smaller but yet significant increases in leaf dry weight compared to R_1_ plants, accompanied by a decrease in leaf area and, consequently, by a decrease in SLA (Table 1).

#### 2.2.2. Effects of Prolonged Recovery after First Drought and Recovery after Third Drought on Cell Wall Composition in WT and *flacca*

**Figure 7:**
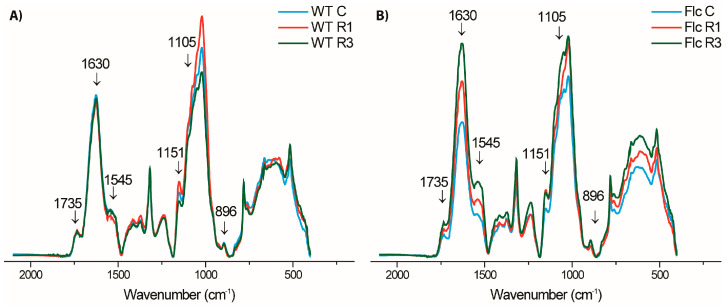
FTIR spectra of the cell walls of WT (**A**) and *flacca* (**B**) tomato leaves at the end of the experiment. R_1_ represents plants exposed to the 1st drought cycle and then optimally watered for the next 15 days. R_3_ represents 3-days re-watered plants after the 3rd drought cycle, while C represents respective control plants.

Overlaid WT and *flacca* tomato leaves’ cell walls’ FTIR spectra are shown in Figure 7 for the control plants, the plants after prolonged recovery and the plants after three drought cycles in the region of 800–1800 cm^−1^. The bands related to cellulose, such as symmetric CH_2_ vibration at 1436 cm^−1^, CH_2_ bending vibration at 1370 cm^−1^, CH_2_ wagging vibration at 1317 cm^−1^, O–C–O asymmetric stretching glycosidic link vibration at 1151 cm^−1^ and C–O stretching vibration at 1105 cm^−1^ were found in all cell wall samples. The bands at 1735 cm^−1^ (C=O stretching vibration of alkyl ester), 1635 cm^−1^ (COO– antisymmetric stretching vibration of polygalacturonic acid), 1420 cm^−1^ (COO stretching, acids), 1240 cm^−1^ (C–O stretching) are characteristic for pectin, while the band at 1517 cm^−1^ is characteristic for lignin. The bands at 1147 cm^−1^ (O–C–O asymmetric stretching glycosidic link) and 1071 cm^−1^ (C–O stretching, C–C stretching) are typical for xyloglucan, and the band at 896 cm^−1^ is related to cellulose, hemicellulose and pectin.

**Figure 8:**
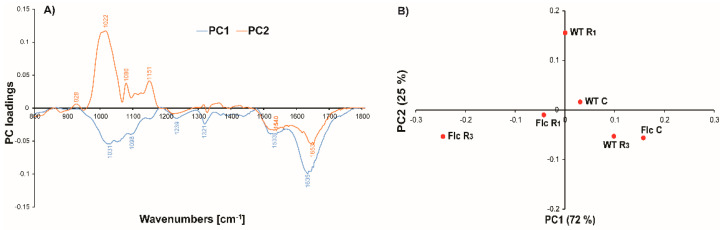
Graphical representation of (**A**) loadings and (**B**) scores for the first two PCs obtained using the spectral region 800–1800 cm^−1^ of tomato samples.

The effect of prolonged recovery, and/or three drought cycles on the content of specific components of the cell wall in the WT and *flacca* mutant leaves of tomato obtained from the FTIR spectroscopy was analyzed using principal component analyses (PCA). PCA was applied on a spectral range (800–1800 cm^−1^) where the first principal component absorbs 72% of the total variance (PC1), while the second component (PC2) absorbs 25% (Figure 8). PC1 negatively correlates with the following wavenumbers at 1031 cm^−1^, 1098 cm^−1^, 1239 cm^−1^, 1321 cm^−1^, 1533 cm^−1^ and 1635 cm^−1^. PC2 positively correlates with wavenumbers at 928 cm^−1^, 1022 cm^−1^, 1080 cm^−1^ and 1151 cm^−1^, and negatively correlates with wavenumbers at 1540 cm^−1^ and 1653 cm^−1^. Based on the values of scores and loadings in PC2 it can be seen that absorption bands characteristic for cellulose and xyloglucan polysaccharides are the most intensive in WT R_1_ in comparison to WT R_3_ and respective controls. On the other hand, intensities for the bands at 1540 and 1635 cm^−1^ (assigned to pectin and proteins) are negatively correlated with PC2. According to scores and loadings for PC1, bands characteristic for cellulose, pectin and lignin are the most pronounced in *flacca* R_3_ and less in *flacca* control plants. The results obtained demonstrate that plants after three drought episodes have elevated contents of cellulose, pectin and lignin compared to *flacca* plants after prolonged recovery and to corresponding controls. According to FTIR spectra analyses, the content of pectin in WT plants remains unchanged between control plants and plants with different drought history and recovery periods. However, in *flacca* plants, drought induced pectin accumulation, with the most prominent increase in plants after three drought cycles. On the other hand, in WT plants, the relative intensity of bands characteristic for cellulose and xyloglucan was the highest in plants after prolonged recovery. Additionally, the content of these polysaccharides was elevated in plants after three drought episodes compared to control plants. Contrarily, in *flacca* plants, three drought cycles caused the greatest accumulation of cellulose, hemicellulose and xyloglucan, while in the prolonged recovery a slight increase in their accumulation was observed in comparison to control plants.

## 3. Discussion

### 3.1. Low ABA Differences Result in flacca Traits

ABA plays a pivotal role in regulating stomatal conductance and transpiration rate and, finally, in developing a plant’s drought tolerance [52]. In our experiment, in comparison to the parental line, leaf ABA content in *flacca* is reduced by around 25% and exhibits a phenotype (smaller whole plants, shorter stems, decreased leaf area, opened stomata, wilted leaves) that is similar to other ABA-deficient tomato mutants described in the literature [49,53,54,55,56,57,58,59]. Numerous reports have shown that drought and recovery period affect ABA`s status in tomato leaves [29,32,60].

Despite differences in constitutive ABA content, the exposure of plants to water deficit in all three drought cycles provoked ABA accumulation in both genotypes, though to a higher extent in WT (Figure 1). While the drought-induced ABA levels were restored during re-watering in WT in all cycles, in *flacca* a similar trend was observed only in the 2nd drought episode. We suppose that some form of ABA conjugates could dominate at that particular stage as a backup for potential upcoming drought [61].

The results obtained could be explained as an ABA-induced “after effect”, as it could control stomatal conductance and transpiration rate in the early recovery phase [62]. This ABA-induced “after effect” could be considered as one of the safety mechanisms, which provides plants’ fast recovery after drought stress [63,64].

Our result on the up-regulation of the *NCED1* gene under water deprivation is in line with the observed ABA accumulation in tomato leaves (Figure 6). The up-regulation of *NCEDs* genes under drought has been demonstrated elsewhere [61,62,65,66]. We assume that the more pronounced expression of the *NCED1* gene in WT observed in the 3rd drought cycle was mostly due to its de novo biosynthesis, while in the 1st and 2nd cycles the ABA was synthesized from its precursors and conjugates. It has been recently shown that, under stress conditions, ROS are involved also in non-enzymatic conversion of ABA precursors to ABA, which might also contribute to the elevated ABA content in the ABA-deficient mutants [67,68]. However, ABA content and transcription intensity of the ABA-biosynthesis-related genes may not always be in correlation, as the level of ABA depends also on ABA`s redistribution within the leaf and between root and shoot [61,69,70].

Considering leaf water potential Ψ, the first drought cycle had a much greater impact on WT than on mutant plants, which is following a greater proline increase in WT compared to *flacca* mutant (Figure 3 and Figure 4). Contrarily, in the 2nd and 3rd drought cycles, an opposite trend was observed in leaf water potential in *flacca*. Moreover, following every drought cycle, the differences in Ψ between drought and recovery plants in both genotypes became smaller [71].

### 3.2. Stomatal Sensitivity Increases with Exposure to Recurrent Drought

Stomatal closure is the fastest physiological response among adaptive mechanisms that prevents water loss in plants under conditions of soil water limitation.

When WT tomato Ailsa Craig cv. plants were subjected to three drought cycles, a percentage of drought-inducible ABA content progressively decreased from the 1st to 3rd cycle (9–14%), while stomata almost closed in the 3rd cycle (g_s_ 40 μmolm^−2^ s^−1^). Under water deficit, though, stomata partly closed even in *flacca*, and transpiration rates were maintained higher in all three cycles in comparison to WT (two to five times higher g_s_ in *flacca*). However, *flacca’s* stomatal aperture was also the most responsive to water deficit in the 3rd cycle in spite of differences in gs between genotypes throughout the experiment (Figure 2). Stomatal closure is mainly determined by the ABA level as a part of chemical signaling [72,73] and, also by hydraulic factors, leaf water potential (Ψ) [74]. Both chemical and hydraulic drought-induced changes were the least in the 3rd cycle where the most efficient stomatal closure was observed. Similar trends in WT and *flacca* imply a possible role of an alternative mechanism that regulates stomata under a recurrent drought. We propose that exposure to a recurrent drought triggers the acquisition of stomatal sensitivity to ABA and Ψ, the alternative factor playing a role in the regulation of stomatal conductance and, thus, in stress memory. The results imply that the ABA level required to trigger efficient stomatal closure must exceed a certain threshold. We showed here a different relationship between ABA, Ψ and stomatal conductance in WT and *flacca*, which implies a hydraulic regulative mechanism of stomatal closure to be preferential in *flacca*, opposite to the dominant ABA-dependent pathway revealed in WT (Figure 1, Figure 2 and Figure 3). The drought-induced fall in leaf water potential in both WT and *flacca* was fully recovered only after the first drought episode, which, in the course of the experiment, progressively led to the establishment of new lower leaf potential. This is in line with recent studies on the effects of developmental and environmental factors on the ABA-acquired stomatal sensitivity in the rosette plant Arabidopsis, which showed that both factors, ontogenic stage of leaf and a relative humidity in the vicinity of a leaf, determined a differential stomatal sensitivity to ABA [75].

### 3.3. Proline Accumulation Decreases with Repeating Drought Cycles

The accumulation of osmolytes under drought is considered as a key protective mechanism against water stress in plant cells. In our previous work with the same tomato genotypes, we demonstrated that the extent of drought-induced accumulation of proline was similar in WT and *flacca*, irrespective of differences in their constitutive ABA levels [51]. However, when plants were grown under higher light intensity, drought induced more than three times higher accumulation of proline in WT than in *flacca*. The opposite trend was observed in *sitiens* tomato mutant where drought negatively affected proline content, while in WT plants proline content increased [76]. On the contrary, salt stress induced higher proline accumulation in *sitiens* compared to WT [56]. Higher proline accumulation under drought was recognized as one of the traits also noticed in tolerant wheat and *Axonopus compressus* grass cultivars in comparison to respective sensitive varieties [77,78]. Besides its widely accepted osmoprotective role, proline is also considered as one of the signaling molecules, and as a symptom of osmotic stress, rather than the adaptation process. Comparison of the dynamics of proline disappearance during the re-watering period in *flacca* (Figure 4) with the results from our previous paper performed under the same conditions except for growth light intensity influence [51], might indicate a role of light and photosynthesis in the drought-induced changes of proline metabolism [79].

In our experiment, we observed the most prominent proline accumulation in both genotypes in the 1st drought cycle, while after the 2nd and 3rd, plants developed weaker responses, with recovery to the control values within 3 days of re-watering. Similarly, Leufen and co-workers [80] obtained the same trend in proline accumulation in sugar beet exposed to recurrent drought. The opposite was observed in coffee and the baru tree with the largest increase in proline observed after the 3rd drought cycle [9,81]. While in WT Ψ decreased to the same extent in all three cycles, in *flacca*, the reduction in Ψ was significantly higher in the 2nd and 3rd drought cycles. As a progressive decrease in Ψ was observed during re-watering in both genotypes, it can be concluded that the capacity of osmolyte accumulation was not enough to prevent a drought-induced decrease in leaf water potential during repeated drought cycles. Moreover, we observed no correlation between ABA and proline accumulation induced by drought that implied that the activation of proline biosynthesis was not an ABA-mediated response to drought as reported elsewhere [82,83,84]. As in our previous article, we here supposed the alternative role to osmoprotection of proline in drought, that is its involvement in cell wall stiffening [51,76]. Our hypothesis is that proline’s role in cell wall fortification via (hydroxyl)proline-rich proteins, is controlled by light through the supply of carbohydrates from photosynthesis, and that low light production of photosynthates and hydroxycinnamates limits the cell wall formation and stiffening.

### 3.4. Drought-Induced Oxidative Stress

Drought-induced changes in the contents of the reduced or oxidized form of ascorbate indicate a disturbance in cellular redox homeostasis, which may be a result either of the activation of the ROS signaling pathway and accompanied antioxidative defense or the increased risk from oxidative damage due to the excessive accumulation of ROS [12]. Providing the ascorbate is the most abundant non-enzymatic antioxidant in the plant cell, and that its concentration is easily determined, it is often used in plant stress physiology studies to evaluate the extent of oxidative stress and antioxidative response [85]. However, the interpretation of stress-induced changes in a reduced or oxidized form of ascorbate is rather ambiguous due to the dual function of ROS [86,87]. In our study, drought induced the ascorbate oxidation only in *flacca* but not in WT, implicating a higher sensitivity of *flacca* to water deficit stress (Figure 5). A simultaneous stimulation of ascorbate biosynthesis in drought upon re-watering during the 2nd and 3rd cycle contributed to the higher redox state of ascorbate in mutants that experienced stress. Though the total ascorbate pool as well as the ascorbate redox state in leaves of both genotypes varied during plant development, a constitutively higher ascorbate content was observed in WT compared to *flacca* throughout the experiment. A constitutively higher ascorbate content was also observed in the Ailsa Craig cv. tomato cultivar compared to the *flacca* mutant [88]. On the other hand, *notabilis* tomato mutants in comparison to WT possess a similar ascorbate level [89], as well as *abi4*-insensitive Arabidopsis mutant [90]. Accordingly, the involvement of antioxidative metabolism in water stress memory was recently reported by Lukic et al. [25]. Authors reported that significantly increased levels of antioxidative enzymes under drought that remained elevated over weeks could be linked with better performances in plants subjected to upcoming stress.

Similarly, there are numerous studies reporting considerably elevated dehydroascorbate pull in stressed plants compared to controls, especially in drought-sensitive cultivars [14,91,92]. Sharma and Dubey [93] showed that mild and severe drought induced ascorbate decline in rice roots and shoots, which was accompanied by a decreased ascorbate redox state. Moreover, Hasanagić et al. [94] showed a decreased ascorbate accumulation in tomato due to prolonged drought, while DHA content remained unchanged until 28 days of water deprivation. These findings are also in line with results obtained in barley and rice where tolerant cultivars had a higher ascorbate content in comparison to sensitive cultivars [14,95].

### 3.5. Drought Differentially Affected Growth and Cell Wall Compounds Accumulation in Two Genotypes

Growth parameters were evaluated at the end of the experiment, which enabled us to compare the effect of prolonged recovery and three drought cycles on biomass and leaf area. A decline in leaf area and the dry biomass of WT tomato plants was observed after three successive drought cycles but not after a single drought episode (Table 1). Similarly, Gomes and co-workers [96] showed that exposure to three drought cycles induced a growth inhibition. On the other hand, drought induced a doubled dry weight of leaves accompanied by a significant decrease in SLA in *flacca*. Our results are in line with the observed growth promotion of recovered plants of alfalfa and maize [97,98]. A smaller effect on growth promotion in *flacca* determined after three drought cycles compared to a prolonged recovery might be the result of shorter intermediate re-watering periods after the 2nd and 3rd drought episodes. One can suppose that an acclimation mechanism induced by drought involves the redistribution or overproduction of advantageous moieties such as sugars, organic acids and antioxidant compounds [99,100]. Similar biomass accumulation in recovered experiments suggests that *flacca* developed acclimation to drought stress by changing only morphological parameters. In this case, the elevated leaf area and dry leaf biomass were strengthened after a prolonged recovery in *flacca*, which implies the important role of a recovery period in the development of a specific plant memory as proposed by Xu and co-workers [101]. Such behavior was initiated during drought and established during the re-watering period.

As a major portion of the dry weight of plants comprises cell wall-derived compounds (approximately 50 ± 5 %), we suppose that the drought-induced accumulation of dry biomass obtained in *flacca* is the result of the accumulated photosynthates and their allocation to the cell wall. Additionally, the accumulation of cell wall compounds would lead to leaf thickening, which could explain why *flacca* plants with a similar dry biomass possess different leaf areas and SLA (Table 1). Such morphological changes induced by drought positively affect photosynthetic efficiency due to tightly packing cells [102].

The stimulation of photosynthesis following drought and recovery has also been obtained in other species [98,101]. Alternatively, it has been suggested that the elevated photosynthesis and increased growth were related to restored stomatal conductance parameters compared to control values [101].

Drought-induced cell wall remodeling includes changes in architecture, accumulation, and cross-linking of cellulose and hemicelluloses–xyloglucan polymers [16], thus, cell wall modulation also contributes to drought tolerance development by maintaining the cell turgor and cell wall elasticity [103].

Based on the extensive comparison analysis of FTIR spectra of cell walls isolated from both genotypes (Figure 7), we further discuss the drought-induced changes of a differential abundance of cell wall constituents [104,105,106,107,108,109,110]. Consequently, a different drought history developed a diverse accumulation of cell wall compounds in both genotypes. In the case of WT leaves, the highest abundance of accumulated cellulose, hemicellulose and lignin was observed at the end of the recovery period after the 1st drought episode but not after three drought cycles (Figure 7). On the contrary, the most pronounced changes in the cell walls of *flacca* leaves were observed in recovered plants after three drought cycles. Cellulose, hemicellulose in total and xyloglucan, as a part of the most dominant hemicellulose polysaccharides, were significantly elevated in recovered plants after three drought cycles, as well as lignin polymers. Likewise, the most prominent changes in the cellulose–hemicellulose complex in WT were observed in recovered plants after one drought, while in *flacca*, this was noticed after three drought episodes. Drought-induced cellulose and hemicellulose accumulation contribute to maintaining cell turgor pressure and cell wall mechanical strength and rigidity, which supports cell protection from water deprivation and permitting their continuous growth [111,112].

Increased lignin deposition and up-regulation of enzymes related to its biosynthesis and accumulation under drought conditions were also reported in numerous articles [113,114,115,116]. In this way, lignin prevents water loss from the leaf, thus contributing to drought tolerance [116].

We also demonstrated the drought-induced biosynthesis of pectin, of which the content, as with other analyzed CW compounds, i.e., cellulose, hemicellulose and lignin, accumulated preferentially in *flacca* leaves after three drought cycles. Nonetheless, one and/or three drought episodes in WT plants did not influence pectin content, and it remained unchanged. With respect to water stress, the amount of side chains of pectic polymers significantly increased in drought tolerant cultivars [117]. Interestingly, there are many reports showing drought tolerant cultivars under drought stress accumulate higher amounts of pectin than susceptible cultivars. An increased pectin level in the cell wall from drought recovered plants in comparison to controls was observed in *Nicotiana sylvestris L*. and *H. annuus* leaves, respectively [118,119]. A higher amount of pectin after three drought episodes in recovery emphasizes their role as gelling agents and antidesiccants in maintaining cell wall hydration status during water deprivation [119].

The drought-induced cell wall thickening of water-conducting and supporting tissues [120] would contribute to more efficient turgor maintenance in otherwise wilting *flacca* plants. The tightening and loosening of cell walls accompanied by changes in the cell wall composition are processes tightly related to cell growth and regulated by various stresses [101]. Water stress certainly provoked cell wall component accumulation and additional cross-linking, which steers towards its fortification, preventing further transpiration and loss of water.

However, cell wall thickening presumably increasing with each subsequent drought cycle may create some kind of physiological memory and, consequently, plants’ higher drought tolerance. Taken together, the accumulation of the aforementioned cell wall components being the most evident in *flacca* after three drought cycles implies that the drought acclimation mechanism was driven through morphological changes, and that prior drought cycles poorly contribute to drought tolerance; rather it is the duration of re-watering periods that are more important.

## 4. Materials and Methods

### 4.1. Plant Material and Experimental Setup

**Scheme 1:**
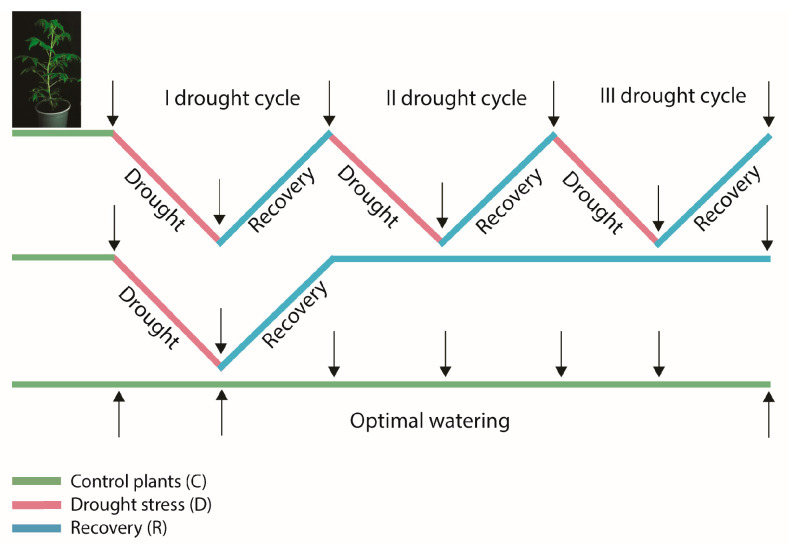
Diagram of experimental design. Plants from both genotypes were submitted to repeated drought stress (red line), followed by recovery period (blue line). At the same time, set of plants were submitted to one drought episode (red lines) and then optimally watered (blue line). Control plants were optimally watered until the end of the experiment (green line).

Wild type (WT) and *flacca* mutant tomato (*Lycopersicon esculentum* Mill. cv. Ailsa Craig) seeds were germinated in pots containing commercial substrate Klasman Potgrond H. Following the phase of four developed leaves, plants were transferred to larger pots (a depth of 24 cm). Plants were grown under controlled conditions with a light intensity of 250 μmol m^−2^ s^−1^, photoperiod 14/10 h (day/night), day/night temperature of 26/17 °C, and 50% relative humidity. Volumetric soil water content (SWC) was continuously maintained at 38 ± 2%. In the phase of 6 leaves, plants from both genotypes were transferred to a light intensity of 800 μmol m^−2^ s^−1^ with the same conditions of photoperiod, temperature, and humidity. After 4 days, plants (start) were divided into three groups. The first group was regularly irrigated during the whole experiment (control plants, C), the second was exposed to just one drought episode (D) and then optimally irrigated (R), while the third was subjected to three drought cycles (D) with recovery periods (R) of three days after each drought. Plants subjected to water deficit were not irrigated until reaching 11 ± 2% moisture in the substrate. Recovered plants from all drought cycles achieved SWC of 38 ± 2% immediately after the first day of the re-watering (Scheme 1).

For each treatment, four biological replicates of both genotypes were prepared. All of the samplings for biochemical measurements and measurements of stomatal conductance and relative water potential were performed using the fourth fully expanded leaf. Sampled leaves were immediately frozen in liquid nitrogen and stored at −80 °C for further analysis.

### 4.2. Measurements of Morphological and Physiological Parameters

Volumetric soil water content (SWC) was measured using the Theta probe (Delta-T, Cambridge, UK), and stomatal conductance (g_s_) was measured by porometer (AP4; Delta-T). Leaf water potential (Ψ, MPa) was carried out with a Scholander-type pressure bomb (Soil Moisture Equipment Corp., Santa Barbara, CA, USA). Fresh and dry weight of both, WT and *flacca* genotypes was determined upon all three drought episodes and after 3-days of recovery period, in treatment, as well as in control plants. Total leaf area (LA) was conducted by LI-3100 areameter (LI-COR, Lincoln, NE, USA), and specific leaf area was calculated using the equation: SLA = Leaf area/DW. All measurements were performed with four different plants per genotype and treatment.

### 4.3. Extraction and Analysis of Abscisic Acid Content

Determination of abscisic acid (ABA) content in the tomato leaves was performed as described in Živanović et al., 2020 [51]. ABA concentration was measured using indirect enzyme-linked immunosorbent assay (ELISA) with MAC 252 monoclonal antibody for ABA (John Innes Centre, Colney, Norwich, UK). Plate contents were measured at 405 nm by a microplate reader (Sunrise, Tecan, Switzerland).

### 4.4. Determination of Leaf Proline Content

In order to determine proline content, frozen leaf samples were homogenized in liquid nitrogen, extracted in 3% (*w*/*v*) sulfosalicylic acid and centrifuged at 14,000× *g* for 10 min at 4 °C. The obtained supernatant was mixed with acidic ninhydrin and glacial acetic acid (1:1:1, *v*/*v*/*v*) and incubated for 60 min on 100 °C. The reaction mixture was placed on ice and extracted with toluene (1:1, *v*/*v*). The toluene fraction was used for determination of proline by measuring absorbance at 520 nm, with toluene as blank [121].

### 4.5. Determination of Total Leaf Ascorbate Content and Ascorbate Redox State

The frozen leaf tissues were homogenized in 1.5% meta-phosphoric acid with 2 mM EDTA and centrifuged at 14,000× *g* for 8 min at 4 °C. The reduced form of ascorbate was measured according to Morina et al. [122]. Briefly, ascorbate (Asc) concentration was determined as absorbance decreased at 265 nm after adding one unit of ascorbate oxidase (Sigma-Aldrich, Darmstadt, Germany) in the reaction mixture consisting of 300 mM potassium phosphate buffer (pH 5.5) and sample. Determination of the total ascorbate content was performed according to Vidovic et al. [123] with some modifications. In order to determine total Asc, the samples were diluted 8 times and incubated with 2.5 U ascorbate oxidase in potassium phosphate buffer (pH 4.5) for 1 min to complete Asc oxidation. After that, reaction mixture was treated with potassium hydroxide to achieve pH 8 and immediately derivatized with ortho-phenylenediamine (o-PDA) for 10 min in the dark. Reaction was stopped with 85% H_3_PO_4_ and samples obtained were loaded on a reversed-phase C18 column (5.0 µm, 250 × 4.6 mm Luna C18 (2); Phenomenex Ltd., Torrance, CA, USA) using the Shimadzu LC-20AB Prominence liquid chromatography (Shimadzu, Kyoto, Japan). The elution gradient was formed with 20 mM potassium phosphate buffer, pH 6.5 (solvent A) and methanol (solvent B): 0–6 min, 30% solution B (isocratic conditions); 6.00–6.01 min, 55% solution B (isocratic conditions); 6.01–13.0 min, 55–30% solution B (linear gradient) at a flow rate of 0.8 mLmin−1. The fluorescence intensity of o-PDA-DHA derivative was measured at excitation and emission wavelengths of 360 and 430 nm using RF-10-AXL, fluorescence detector (Prominence, Shimadzu, Japan).

### 4.6. RNA Isolation and Real Time PCR

RNA was isolated by TRIzol™ Reagent (Invitrogene, Waltham, MA, USA) according to manufacturer’s protocol. RNA was quantified with a NanoDrop spectrophotometer (IMPLENP300, Munich, Germany), and its quality was checked by electrophoresis on 1.5% agarose gel. To remove traces of DNA contamination, RNA was treated with DNase I (Thermo Fisher Scientific, Waltham, MA, USA) at 37 °C for 10 min, according to the manufacturer’s protocol. Synthesis of cDNAs from 1 µg of total RNA was carried out in the reverse transcription reaction (RT), according to the manufacturer`s protocol RevertAid First Strand cDNA Synthesis Kit (Thermo Scientific™, Waltham, MA, USA).

Expression of *NCED1* gene, which is involved in abscisic acid biosynthesis, was evaluated by quantitative real time PCR using SYBR green in 7500 Real Time PCR System (Applied Biosystems, Waltham, MA, USA). A total of 10 µL reaction mixture contained 1 μL of RT reaction product, appropriate forward and reverse primers and Maxima SYBR Green/Rox qPCR Master Mix (Thermo Fisher Scientific, Waltham, MA, USA). The forward primer 5′AGGCAACAGTGAAACTTCCATCAAG3′ and reverse primer 3′TCCATTAAAGAGGATATTACCGGGGAC5′ were used for *NCED1* amplification (GenBank^TM^Accession No. Z97215). Thermal cycling conditions for qRT-PCR included: initial denaturation on 95 °C, then 40 cycles of denaturation (95 °C for 30 s), annealing (55 °C for 30 s) and extension (72 °C for 30 s). The absence of primer-dimer formation was checked in no-template controls. In an amplification reaction, the point in the cycle at which the target DNA has amplified enough so that its fluorescence reaches fixed threshold above the background fluorescence is called the Ct value of the sample. The expression level of the tested gene was normalized to the housekeeping gene actine and calculated relative to WT start control according to the ∆∆Ct method. The results are presented as log2 transformation of fold changes (log2FC). For each sample, qRT-PCR was performed in triplicate [124].

### 4.7. Cell Wall Isolation and Purification

The plant cell wall was isolated according to the procedure described in Simonović-Radosavljević et al. [125]. The dried plant leaves were powdered with mortar and pestle and extracted in 80% methanol (1/8, *w*/*v*) with shaking for 60 min at room temperature. The homogenate was centrifuged at 1000× *g* for 20 min at room temperature and pellet was washed two times with 80% methanol. The pellet was resuspended in 1 M NaCl with 0.5% Triton ×100 and centrifuged at 1000× *g* for 20 min at room temperature. The pellet was re-washed with distilled water, once with absolute methanol and twice with acetone. The purified cell wall obtained was dried and additionally powdered in Mixer Mill MM 400 (Retsch) and further used for structural analyses.

### 4.8. FTIR Spectroscopy

The Fourier-transform infrared (FTIR) spectra of the extracted cell wall materials were recorded using a Perkin Elmer Spectrum Two equipped with the Universal ATR accessory. The spectrum of each powder sample was collected in the range of 4000–400 cm^−1^ with 200 scans and a spectral resolution of 4 cm^−1^. Baseline correction was performed using the trial Spectra Gryph software (https://www.effemm2.de/spectragryph/index.html, accessed on 17 March 2021).

### 4.9. Statistical Analysis

Three-way ANOVA was used to reveal the effects of genotypes, number of drought cycles and water regimes and their interactions on g_s_, ψ, morphological parameters, ABA, proline and ascorbate contents. Tukey post hoc test was used for specific comparisons among experimental groups and significance threshold value was set at *p* ≤ 0.05.

Welch t-test was used to determine differences in *NCED1* gene expression. The experimental data processing was conducted using software package Statistica 8.0.

In order to investigate the similarities between the samples, principal component analyses (PCA) was performed on the FTIR spectra, in the region between 1800 and 800 cm^−1^ [126]. PCA decomposes the variation of matrix **X** into score matrix (**T**), loading matrix (**P**) and residual matrix (**E**): **T = TP***^T^* **+ E**. The similarity of the samples can be visualized by reducing the dimensionality of the space, i.e., their projection on a subspace defined with a smaller number of dimensions (principal components). If the first two principal components absorb a sufficient percentage of the total variations, then the samples can be projected onto a plane spanned by the vectors of these principal components, which facilitates the visual identification of pattern similarities.

No preprocessing of data was required. The software Unscrambler X 10.4 (Camo Analytics, Oslo, Norway) was used for multivariate analysis.

## 5. Conclusions

In this work, we demonstrated that analyzed tomato genotypes (*flacca* mutant and its parental line Ailsa Craig cv.) developed different drought tolerant strategies depending on their constitutive ABA levels. A failure of the efficient stomatal closure mediated by drought-inducible ABA accompanied by lower leaf potential and transient oxidative stress are compensated by a double increase in the dry weight of *flacca* leaves in plants that experienced prolonged recovery. Drought induced more pronounced stomatal closure, ABA biosynthesis and proline accumulation in WT compared to the mutant, confirming the importance of stomata in water loss prevention in WT. The most efficient stomatal response observed in the 3rd drought cycle in WT, which did not correlate with ABA increase, implies that repeating drought cycles trigger the acquisition of stomatal sensitivity to a chemical and/or a hydraulic signal. On the other hand, the osmoprotective role of proline was not pertinent for developing drought stress memory, considering that the drought-induced accumulation of proline was dramatically decreased with repeating drought cycles.

We hypothesize that under certain conditions imposed by lower ABA content (*flacca*) and saturating, high growing light, modification of the cell wall occurred. Strengthening of the vascular tissue and cell wall capacity of water storage are traits favored by the enhancement of networking between cellulose, hemicellulose, pectin and hydroxyproline protein enrichment, and may present a target for future improvement of drought tolerance.

## Figures and Tables

**Table 1:** Leaf, stem and total shot fresh (FW) and dry weight (DW) per plant, leaf area and specific leaf area (SLA) of WT and *flacca* tomato genotypes at the end of the experiment. R_1_ represents plants exposed to the 1st drought cycle and then optimally watered for the next 15 days. R_3_ represents 3-days re-watered plants after the 3rd drought cycle, while C represents respective control plants. Values are presented as means ± SE (*n* = 4). Different letters denote significant differences between means in every genotype separately, according to Tukey HSD post hoc test *p* ≤ 0.05.

**WT**			
	**C**	**R_1_**	**R_3_**
Leaves FW (g)	31.86 ± 0.94 b	40.75 ± 1.97 c	19.89 ± 1.63 a
Stem FW (g)	26.14 ± 0.59 ab	23.84 ± 2.63 a	33.47 ± 1.65 b
Plant FW (g)	58.01 ± 1.53 a	64.59 ± 4.60 a	53.37 ± 3.3 a
Leaves DW (g)	2.08 ± 0.02 b	2.36 ± 0.05 c	1.63 ± 0.07 a
Stem DW (g)	2.10 ± 0.06 a	2.17 ± 0.30 a	1.70 ± 0.19 a
Plant DW (g)	5.78 ± 0.11 ab	6.09 ± 0.41 b	4.50 ± 0.36 a
LA (cm^2^)	1019. 9 ± 63.1 ab	1137.9 ± 5.9 b	880.8 ± 13.9 a
SLA (cm^2^ g^−1^DW)	490.6 ± 26.8 a	483.2 ± 7.7 a	541.5 ± 16.1 a
*flacca*			
	C	R_1_	R_3_
Leaves FW (g)	27.26 ± 0.63 a	38.49 ± 2.07 b	27.08 ± 3.69 a
Stem FW (g)	15.84 ± 0.60 b	15.55 ± 0.71 ab	13.01 ± 0.50 a
Plant FW (g)	43.10 ± 0.1 ab	54.1 ± 1.6 b	40.1 ± 4.2 a
Leaves DW (g)	1.13 ± 0.01 a	2.23 ± 0.01 b	1.97 ± 0.16 b
Stem DW (g)	1.05 ± 0.02 a	1.56 ± 0.01 b	0.88 ± 0.09 a
Plant DW (g)	2.91 ± 0.01 a	5.28 ± 0.02 b	4.16 ± 0.46 b
LA (cm^2^)	630.1 ± 3.8 a	963.9 ± 8.7 b	544.1 ± 41.7 a
SLA (cm^2^ g^−1^DW)	557.7 ± 3.8 c	433.1 ± 3.2 b	276.7 ± 1.1 a

## Supplementary Materials

The following are available online at https://www.mdpi.com/article/10.3390/plants10112308/s1, Table S1: Statistical analysis of the results (three-way ANOVA) to test the influence of the treatment, number of drought cycles and two genotypes (WT and *flacca*), and their interactions on the amount of ABA, stomatal conductance (g_s_) and relative water potential (Ψ) in tomato leaves; Table S2: Statistical analysis of the results (three-way ANOVA) to test the influence of the treatment, number of drought cycles and two genotypes (WT and *flacca*) and their interactions on the proline content, total ascorbate content and ascorbate redox state in tomato leaves.
