# Supplementary material for: Differential Response of Two Tomato Genotypes, Wild Type cv. Ailsa Craig and Its ABA-Deficient Mutant flacca to Short-Termed Drought Cycles
Source: Plants (Basel). 2021 Oct 27;10(11):2308. doi: 10.3390/plants10112308 (PMC8617711; doi:10.3390/plants10112308)
Supplement: Supplementary file 1 [file plants-10-02308-s001.zip › plants-1424301-supplementary.pdf]

Supplementary Table S1. Statistical analysis of the results (three-way ANOVA) to test the influence of the treatment, number of drought cycles and two genotypes (WT and *flacca*) and their interactions on the amount of ABA, stomatal conductance (gs) and relative water potential (Ψ) in tomato leaves.

Trait	Source of variation	df	F	*p*
ABA	Genotype	1	140.79	0.000000
	Cycles	2	0.21	0.807973
	Treatment	2	73.31	0.000000
	Genotype x Cycles	2	5.07	0.008248
	Genotype x Treatment	2	23.51	0.000000
	Cycles x Treatment	4	0.63	0.641365
	Genotype x Cycles x Treatment	4	2.10	0.087148
gs	Genotype	1	587.30	0.000000
	Cycles	2	60.83	0.000000
	Treatment	2	113.57	0.000000
	Genotype x Cycles	2	15.76	0.000001
	Genotype x Treatment	2	50.90	0.000000
	Cycles x Treatment	4	88.88	0.000000
	Genotype x Cycles x Treatment	4	51.39	0.000000
Ψ	Genotype	1	11.28	0.001255
	Cycles	2	90.07	0.000000
	Treatment	2	154.89	0.000000
	Genotype x Cycles	2	24.75	0.000000
	Genotype x Treatment	2	1.78	0.176347
	Cycles x Treatment	4	6.08	0.000284
	Genotype x Cycles x Treatment	4	11.25	0.000000

Supplementary Table S2. Statistical analysis of the results (three-way ANOVA) to test the influence of the treatment, number of drought cycles and two genotypes (WT and *flacca*) and their interactions on the proline content, total ascorbate content and ascorbate redox state in tomato leaves.

Trait	Source of variation	df	F	*p*
**Proline**	Genotype	1	84.97	0.000000
	Cycles	2	213.19	0.000000
	Treatment	2	320.53	0.000000
	Genotype x Cycles	2	112.09	0.000000
	Genotype x Treatment	2	114.08	0.000000
	Cycles x Treatment	4	222.32	0.000000
	Genotype x Cycles x Treatment	4	99.92	0.000000
**Total Ascorbate**	Genotype	1	44.27	0.000000
	Cycles	2	76.90	0.000000
	Treatment	2	27.14	0.000000
	Genotype x Cycles	2	23.90	0.000000
	Genotype x Treatment	2	22.16	0.000000
	Cycles x Treatment	4	8.29	0.000010
	Genotype x Cycles x Treatment	4	1.32	0.267438
**Ascorbate redox state**	Genotype	1	0.01	0.911552
	Cycles	2	13.09	0.000010
	Treatment	2	17.11	0.000001
	Genotype x Cycles	2	30.29	0.000000
	Genotype x Treatment	2	16.69	0.000001
	Cycles x Treatment	4	9.79	0.000001
	Genotype x Cycles x Treatment	4	3.39	0.012384
